# Applying *C. elegans* to the Industrial Drug Discovery Process to Slow Aging

**DOI:** 10.3389/fragi.2021.740582

**Published:** 2021-10-19

**Authors:** David Weinkove, Giulia Zavagno

**Affiliations:** ^1^ Department of Biosciences, Durham University, Durham, United Kingdom; ^2^ Magnitude Biosciences Ltd., NETpark Plexus, Sedgefield, United Kingdom

**Keywords:** aging, *C. elegans*, drug discovery, efficacy, toxicity, industry, regulatory

## Abstract

The increase in our molecular understanding of the biology of aging, coupled with a recent surge in investment, has led to the formation of several companies developing pharmaceuticals to slow aging. Research using the tiny nematode worm *Caenorhabditis elegans* was the first to show that mutations in single genes can extend lifespan, and subsequent research has shown that this model organism is uniquely suited to testing interventions to slow aging. Yet, with a few notable exceptions, *C. elegans* is not in the standard toolkit of longevity companies. Here we discuss the paths to overcome the barriers to using *C. elegans* in industrial drug discovery. We address the predictive power of *C. elegans* for human aging, how *C. elegans* research can be applied to specific challenges in the typical drug discovery pipeline, and how standardised and quantitative assays will help *C. elegans* fulfil its potential in the biotech and pharmaceutical industry. We argue that correct application of this model and its knowledge base will significantly accelerate progress to slow human aging.

## Introduction

As we start to understand aging on a molecular level, there is an increasing drive to find pharmaceutical interventions that slow aging. The case to do so is strong: slowing aging would delay the onset of many chronic diseases, for most of which we have no effective therapy (e.g. Alzheimer’s disease). Furthermore, studies suggest that two current pharmaceuticals, metformin—a diabetes drug, and rapamycin—an immunosuppressant, could be repurposed to slow aging (Soukas et al., 2019; Selvarani et al., 2021). A recent boom in biotech investment has resulted in several companies raising huge sums of money to develop new drugs to tackle aging. Yet, these companies face tremendous challenges. In this Perspective, we will discuss these challenges and how the nematode *Caenorhabditis elegans* can assist research in industrial drug discovery. *C. elegans* has a special place in the history of aging research because it was the first animal where single gene mutations resulted in a substantial increase in lifespan. Yet despite this success, *C. elegans* is not used routinely in industrial research and development. We will discuss why not and how careful application of *C. elegans* research could help address the key challenges of drug discovery for aging.

## Finding out Quickly and Effectively if the Drug Slows Aging

The primary question for any drug candidate is whether it slows aging, and thus compounds need to be tested for efficacy in a biological model that ages as soon as possible in the development pipeline. Measuring aging in cultured mammalian cells is almost impossible. The closest model available is measuring senescence in primary human fibroblasts (Hayflick and Moorhead 1961). Aging can be accelerated in these cells by inducing molecular damage (Petrova et al., 2016). However, these experiments are limited by availability of donor tissue, inconsistency of the source (every person is different—genetically, age, medical history), and variations in methodology (Smith et al., 2002). Proceeding directly to testing compounds for aging in mice is slow and expensive, as mice have a lifespan of about 3 years. Aging can be measured at earlier ages in the mouse but there is not a consensus on the age range to use, and experiments last a minimum of several months (Jackson et al., 2017). Thus, *C. elegans,* with a 2–3 weeks lifespan, is an attractive model to efficiently test compounds for their ability to slow aging before proceeding to mice. Large populations can be tested, which helps deal with the high variation in aging between individuals found in all animal models (Petrascheck and Miller 2017).

## Regulatory Approval—is the Drug Safe?

All drugs have to be shown to be safe, but for a drug to slow aging there are specific considerations. The side effects of approved drugs are tolerated by regulatory authorities because the benefit outweighs the risk. In the case of slowing aging, an essentially healthy person could be exposed to a drug for decades, so the drug must show impeccable safety to stand a chance of approval. Currently the FDA and other authorities do not recognise aging as an indication, and thus cannot approve a drug to treat it, even if the drug is shown to be safe and effective. However, this position may change. Many are looking to the Targeting Aging with Metformin trial (TAME), approved by the FDA (Barzilai et al., 2016). If successful, this trial may lead to regulatory changes. For now, most drug developers are instead targeting specific age-related diseases with the hope that these drugs will also slow aging in general.

Before regulators will allow progression to clinical trials, there must be a preclinical research phase with standard requirements (European Medicines Agency 2008; Federal Drug Administration 2010). As part of this phase, drugs must be tested for safety in two mammalian species. This expensive step is bound by regulation that also makes experiments time-consuming to arrange. It would be desirable to eliminate drugs with signs of toxicity before proceeding to this point. Some toxicity work can be performed in *in vitro* cell culture but these methods do not reveal toxicity to the whole organism and in particular, do not reveal developmental and reproductive toxicity, or toxicity from prolonged chronic exposure. *C. elegans* research has no regulatory restrictions and acute, developmental and reproductive toxicity can be studied in the same experiment (Hunt 2016). Chronic toxicity can be detected in worms as they age. In studies conducted so far, the ranking of toxicity of a series of compounds in *C. elegans* follows results in mammals closely even if the absolute concentrations are not directly comparable (Williams and Dunesbery 1988; Harlow et al., 2016). Thus, trials in *C. elegans* can provide early warnings of safety issues.

## Is *C. elegans* An Appropriate Animal Model?

It is important that the chosen model is appropriate for the specific drug development problem to be solved and that the drug developer can justify the choice to their stakeholders. These stakeholders may include their investors, who are required to support further drug development, and their partners in biotech and pharma who may ultimately license the intellectual property and bring the drug to the clinic. It will also include the regulators, who will examine the results of the R&D process before allowing progression to clinical trials. For *C. elegans*, it is very reasonable to question why a drug that extends the lifespan of an animal that only lives for a few weeks would be expected to slow aging in an animal that lives for several decades. Yet many interventions, either genetic disruption of conserved pathways or treatment with compounds, slow aging in very different model organisms: *C. elegans*, *Drosophila*, mice and even yeast (Johnson et al., 2013; Bhullar and Hubbard 2015; Taormina et al., 2019). For example, metformin and rapamycin extend lifespan in *C. elegans* (Cabreiro et al., 2013; Robida-Stubbs et al., 2013). The insulin/IGF signalling (IIS) pathway was initially discovered in *C. elegans* using genetics screens. Its relevance to humans has been supported by studies showing association of longevity with lower circulating IGF-1 levels or a specific allele of FOXO3, a homologue of the *C. elegans* gene *daf-16* (Suh et al., 2008; Willcox et al., 2008; van der Spoel et al., 2015). These results suggest a common property of interventions that slow aging—that they work through universal properties of cell biology and/or animal physiology. It is yet to be seen if this will be true for all drugs that will ultimately be found to slow human aging. However, it is a good working assumption that if a compound slows aging in *C. elegans* it has a better chance of success than one that does not. Therefore, this information can be crucial in the decision-making process to prioritise drugs for further development and preclinical trials.

Target conservation is an important issue for drug development. *C. elegans* has a similar number of protein-coding genes as humans (approx. 20,000) (Willyard 2018) and many well conserved homologues (Mack et al., 2018). Most proteins involved in cell biology and metabolism have clear homologues in *C. elegans*, albeit often *C. elegans* has fewer isoforms of the same protein. For example, *C. elegans* has only one Class IA PI 3-kinase (AGE-1), whereas humans have three (Kriplani et al., 2015). Conservation can be very high at the amino acid level, particularly in conserved regions such as the active site, but needs to be evaluated on a protein-by-protein basis. Compounds are usually designed to interact with a particular area of a target enzymes and if there is insufficient conservation in the *C. elegans* homologue, this area or the entire protein can be replaced by the human gene using Cas-CRISPR technology (McDiarmid et al., 2018; Vincencio and Cerón 2021).

Scepticism about animal models is prevalent in the field of neurodegenerative disease because there have been some high-profile failures of drugs that produced positive results in lab mammals but failed clinical trials in humans - most notably BACE inhibitors for Alzheimer’s disease (Egan et al., 2018; Panza et al., 2018). These drugs inhibit the enzyme that cleaves the amyloid beta peptide, which can then form amyloid plaques in the brain (Vassar et al., 1999). In Phase III clinical trials, these drugs reduced plaque formation but did not slow cognitive decline. Many believe the underlying hypothesis was wrong, or that the drugs needed to be administered before patients showed symptoms. Regardless, the failure of animal models to predict the failure of these drugs needs to be examined. It does not mean that animal models cannot be used to develop further therapies but companies are attracted to the vastly improved tools developed for performing genetic studies with cultured human cells such as CRISPR and induced pluripotent stem cells. However, unlike *C. elegans*, these models do not age and do not simulate the whole organism context, and thus do not yet replace animal models of aging.

The other advantage of using an animal model is that any side effects of compounds can be seen in the context of multi-organ physiology. The long-lived *C. elegans* IIS mutants show a small reduction in fertility, which in the case of the *age-1(hx546)* mutant is only seen during cycles of starvation and feeding (Walker et al., 2000; Jenkins et al., 2004). Long-lived IIS mutants in flies and mice also show infertility. Thus, the *C. elegans* model is a useful predictor of the whole organism effects of interventions that slow aging. It allows the most promising interventions to be identified at an early stage of drug development and can even reveal methods to avoid side effects—for example by timing the intervention to a later age (Dillin et al., 2002). *C. elegans* research will never be translatable at all aspects of the drug development pipeline but can be very useful at specific stages depending on the project.

## Reproducibility and Standardisation of Data—An Industrial Approach

Industrial drug developers need confidence in the data they use to make key decisions. Academic research produces new biological insights that provide the underlying rationale for drug discovery. However, the workflow in academic research groups is not usually well suited for the systematic testing of lead compounds. Furthermore, there is a known issue of reproducibility between labs. Differences in outcome between labs are often caused by subtle differences in methodology that are not captured in the methods reported in basic research papers. In the field of aging, methodological differences are particularly important because multiple environmental factors can influence the aging and survival of lab animals. Many features of the academic *C. elegans* community ensure some degree of standardisation, such as the distribution of common strains, the ability to freeze strains and the use of common methods, along with a collaborative culture in the field. However, our own experience has found that there are many small differences in methods between labs, such as how media is prepared, how the bacteria used to feed the worms are grown, and the time between making media and using it. Some labs have tried to overcome these issues through publishing extremely detailed methods such sources of reagents including batch numbers (Lithgow et al., 2017).

Another issue that can affect reproducibility is the manual scoring of assays to measure aging in *C. elegans*, such as the standard lifespan assay. Transferring worms from one plate to another and accurately recording the time of death requires skill, and is vulnerable to subjective interpretation. Automation has the potential to provide objective quantifiable results. Automation covers many areas of the process including worm handling, worm observation and downstream data analysis. Several groups have invented automated methods to quantify aging in *C. elegans* (Stroustrup et al., 2013, Xian et al., 2013, Li et al., 2015, Churgin et al., 2017, Letizia et al., 2018, Saberi-Bosari et al., 2018, Benedetto et al., 2019, Rahman et al., 2020, Le et al., 2020, reviewed in Felker et al. (2020), Puchalt et al. (2021). These include The Lifespan Machine, which monitors lifespan of *C. elegans* on Petri dishes in an adapted commercial flat-bed scanner. It has been adopted by the *Caenorhabditis* Interventions Testing Program, an NIH-funded consortium of academic labs that tests compounds for aging effects (Banse et al., 2019). Another technology is the WormMotel (Churgin et al., 2017), which was adopted by the EU ‘Ageing with *elegans*’ consortium. Our group has invented a distributed imaging technology (Tataridas-Pallas et al., 2021) to measure how worms slow down with age while they are on Petri dishes (Zavagno et al., unpublished). This method can detect effects on aging within 7–10 days and produces data on how a compound influences animal movement. As well as providing an objective readout of *C. elegans* aging, we have found another benefit: recording how and where worms are moving on the Petri dish from the beginning of the experiment has shown that small differences in media preparation can influence the initial behaviour of the worms and is likely to affect experimental outcomes. This feedback has allowed us to further standardize our procedures to prepare worms, media and bacteria.

There have been several previous attempts to involve *C. elegans* in pharmaceutical drug discovery. Biotech companies including Nemapharm, Exelixis, Elixir Pharmaceuticals and DevGen formed in the late 1990s using *C. elegans* (Wells 1998; McCarthy 2004, 2005). Many of these companies partnered with, or were acquired by, larger pharma or biotech companies but none produced a sustainable *C. elegans* drug development programme. We argue that the drug discovery landscape has changed considerably over the last 2 decades, with a much larger ecosystem of biotechs involved and a greater role for contract research organisations (CROs). Specialised *C. elegans* CROs provide an easier option to companies that want to engage in *C. elegans* research but cannot invest in establishing an in-house facility, which requires considerable expertise and specialist equipment. Until recently the only outsourcing option was to work with an academic *C. elegans* lab, but in the last few years a small number of companies have been offering *C. elegans* research services (Butlerijs and Braeckmann 2020).

## Taking *C. elegans* Research Further: Understanding of Mechanism of Action and Screening for Derivatives

Once a compound has been shown to slow aging in *C. elegans* in a way that suggests it will be safe, further possibilities of this genetic model open up. The first is to help establish the mechanism of action. Regulators are much more likely to approve compounds if the mechanism of action is understood. It also allows drug developers to make rational decisions about optimising drug design and generating new derivatives. Mechanism of action can be studied in a number of ways in *C. elegans*: for example by measuring effects on gene expression either globally *via* RNAseq, or specifically by measuring downstream targets of known pathways, often by fluorescent reporters in live worms. Further work includes testing the involvement in known pathways or suspected target proteins using mutants or RNA interference. Furthermore, proteotomics, lipodomics and metabolomics are widely used in *C. elegans* research and can illuminate mechanism of action. In fact, there is a wealth of *C. elegans* techniques which cannot be adequately covered in this perspective (Chen et al., 2015; Apfeld and Alper 2019; Kropp et al., 2021). High throughput assessment of aging in *C. elegans* opens the possibility that potential targets and downstream pathways can be identified using genetics or through random mutagenesis screening. Secondly, once an effect is found, it can be developed into an assay with increased throughput, allowing hundreds or thousands of drug derivatives created by medicinal chemistry to be tested for their ability to slow aging. A similar approach has been used to screen libraries of drugs in *C. elegans* to find those that slow aging (Petrascheck and Miller 2017; Butlerijs and Braeckmann 2020) Our opinion is that the possibilities for *C. elegans* to help aging drug discovery has only just begun.

## Conclusion

The development of drugs that slow aging faces considerable challenges. To even be considered for clinical trials, evidence is required that the compound is both effective and incredibly safe over a long period. It also important to find out as much as possible about the mechanism of action. In this perspective we have argued that research with *C. elegans* can assist with several of these challenges before the compound is tested in a mammalian model, thereby improving the chances that a lead compound will pass through later stages of drug development. We think that in the future, as *C. elegans* research is shown to accelerate drug discovery, it will drive further adoption of the technology. In fact, *C. elegans* was recently used to find an existing drug that could be repurposed effectively and had beneficial effects in treating a child with a genetic phosphomannomutase 2 deficiency (Iyer et al., 2019; Perlstein et al., 2021). Inspiring success stories like this one increase the interest in using *C. elegans* for drug discovery.

It is important to stress that *C. elegans* cannot solve all the problems of early-stage drug development and there are several disease states that cannot be well translated to this model. However, whole organism aging does seem to be a field in which translation works well. *C. elegans* must also be used in conjunction with several other distinct technological approaches. A key consideration for anyone aiming to use *C. elegans* in drug development is to identify the major obstacles specific to each pipeline and tailor *C. elegans* research to address them where relevant. There may well be several steps in the pipeline in which *C. elegans* can be applied and the earlier in the pipeline the number of chemical series or lead compounds is reduced, the more time and money can be saved later. Our perspective, from our experience with engaging with industrial end users, is that increasing the interaction between the scientists driving the industrial programme and the *C. elegans* scientists designing the research will lead to better use of this extraordinary model to accelerate progress in longevity drug development.
